# Boosting Vaccine Research: The 16-Year Journey of TRANSVAC Vaccine Infrastructure

**DOI:** 10.3390/vaccines12121446

**Published:** 2024-12-22

**Authors:** William Martin, Catarina Luís, Stefan Jungbluth, Monika Slezak, Frank A. W. Verreck, Holger Spiegel, Carlos A. Guzman, António Roldão, Manuel J. T. Carrondo, Peter Van der Ley, Joaquim Segalés, Hazel M. Dockrell, Mei Mei Ho, Gabriel K. Pedersen, Maria Lawrenz, Ole F. Olesen

**Affiliations:** 1 European Vaccine Initiative (EVI), 69115 Heidelberg, Germany; 2 Biomedical Primate Research Centre (BPRC), 2288 GJ Rijswijk, The Netherlands; 3 Fraunhofer Institute for Molecular Biology and Applied Ecology IME, 52074 Aachen, Germany; 4 Department of Vaccinology and Applied Microbiology, Helmholtz Centre for Infection Research (HZI), 38124 Braunschweig, Germany; 5 iBET—Instituto de Biologia Experimental e Tecnológica, 2781-901 Oeiras, Portugal; 6 Intravacc BV, 3721 MA Bilthoven, The Netherlands; peter.van.der.ley@intravacc.nl; 7 Unitat Mixta d’Investigació IRTA UAB en Sanitat Animal, Centre de Recerca en Sanitat Animal (CReSA), Departament de Sanitat i Anatomia Animals, Facultat de Veterinària, Campus de la Universitat Autònoma de Barcelona, 08193 Bellaterra, Spain; joaquim.segales@irta.cat; 8 Department of Infection Biology, London School of Hygiene & Tropical Medicine, London WC1E 7HT, UK; 9 Medicines and Healthcare Products Regulatory Agency (MHRA), Potters Bar EN6 3QG, UK; mei.ho@mhra.gov.uk; 10 Department of Infectious Disease Immunology, Statens Serum Institut, Artillerivej 5, 2300 Copenhagen, Denmark; 11 Vaccine Formulation Institute (VFI), Plan-les-Ouates, 1228 Geneva, Switzerland

**Keywords:** research infrastructure, vaccine, research and development

## Abstract

TRANSVAC represents a long-running effort to accelerate the development of novel vaccines by integrating institutions from across Europe under a single collaborative framework. This initiative has empowered the global vaccine community since 2009 including contributing toward the development and optimization of vaccine candidates as well as the provision of new adjuvants, research protocols, and technologies. Scientific services were provided in support of 88 different vaccine development projects, and 400 professionals attended TRANSVAC training events on various vaccine-related topics. Here, we review the accomplishments of the TRANSVAC consortia and analyze the continued needs of academic and industrial vaccine developers in Europe. The findings highlight the benefits of coordination across different sectors, both through research infrastructures such as TRANSVAC and other mechanisms, to address the current and future global health challenges and ensure that European vaccine developers have the support required to successfully compete in the global market.

## 1. Introduction

**Figure 1:**
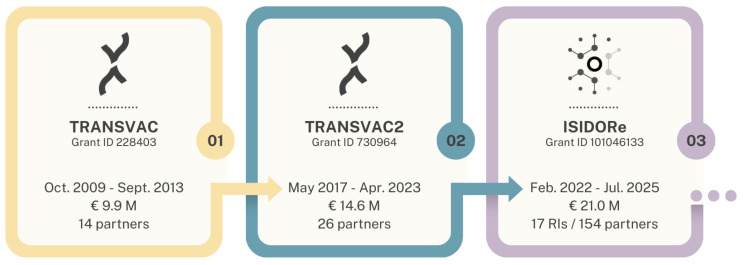
Overview of TRANSVAC projects.

**Figure 2:**
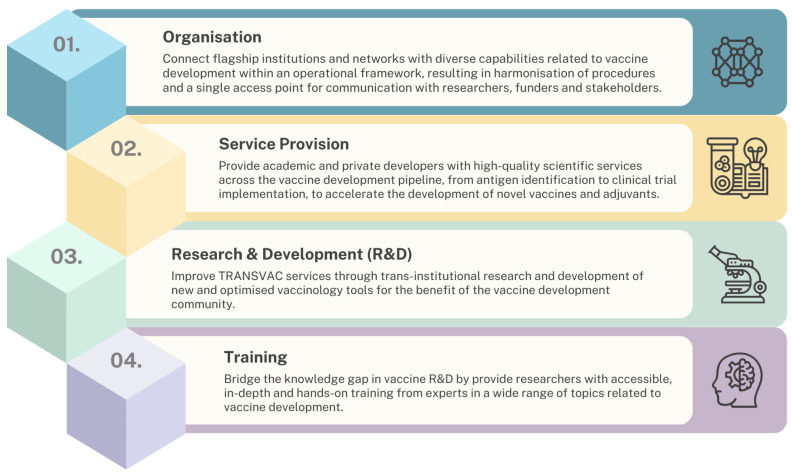
Pillars of the TRANSVAC vaccine infrastructure.

TRANSVAC was established as a vaccine-focused distributed research infrastructure (RI) in 2009, with the first of three successive TRANSVAC projects running until 2013 (Figure 1). In each iteration, TRANSVAC sought to promote vaccine development through its core pillars of organization, service provision, research and development (R&D), and training (Figure 2). Distributed RIs such as TRANSVAC are networks of cross-institutional facilities with complementary technologies and expertise in a particular scientific domain. RIs form a key mechanism for coordination within European scientific communities, providing external researchers with access to relevant services and resources in order to accelerate scientific achievements and promote sustainable research. The earliest TRANSVAC project was coordinated by the European Vaccine Initiative (EVI) and funded by a contribution of EUR 9.9 M from the European Union (EU) through the 7th Framework Program [1]. The first TRANSVAC consortium consisted of 14 institutions including academic institutions, public and private non-profit research institutions, public agencies, and biotechnology companies, with complementary expertise in vaccine development (Table 1). In this initial version of TRANSVAC, the services provided focused on adjuvant formulation, animal models, and sequencing. These activities were provided free of charge at TRANSVAC institutions to applicants that had been selected following a peer review process.

The first TRANSVAC project (henceforth referred to as TRANSVAC1 for clarity) was followed by TRANSVAC2, which operated from 2017 to 2023. TRANSVAC2 had 26 partner institutions (Table 1) and a significantly enlarged scope, with a total budget of EUR 14.6 M (including EUR 4.0 M of earmarked funding provided in early 2020 by the EU in response to the SARS-CoV-2 pandemic). The additional partners expanded the range of TRANSVAC services to include antigen expression platforms, biochemical and structural biology analysis, immunological assays, multi-omics, process scale-up, Good Laboratory Practice (GLP) production as well as support for regulatory and clinical trial processes. Additionally, a single training course provided during TRANSVAC1 was expanded into a full program of 14 training courses organized by TRANSVAC2 partners.

At the end of TRANSVAC2, 20 of the 26 partners continued offering vaccine-related scientific services within the framework of the 3-year project ‘Integrated Services for Infectious Disease Outbreak Research’ (ISIDORe) (https://isidore-project.eu/, accessed on 20 October 2024). This large consortium comprises 154 partner institutions organized into 17 European research infrastructures including TRANSVAC and focuses on improving pandemic preparedness by providing services to researchers and integrating complementary networks under a single framework. The project is funded until July 2025 with a budget of EUR 21.0 M as part of the EC’s Health Emergency Preparedness and Response Authority (HERA) (Table 1).

Many of the gaps and needs that initially prompted the creation of TRANSVAC remain relevant and urgent today [2]. The Coronavirus disease 2019 (COVID-19) pandemic and ensuing public health response demonstrated the importance of a strong and sustainable local vaccine landscape. Effective, first-in-class vaccines were rapidly developed and deployed to approximately 286 million people in the World Health Organization (WHO) European Region in the 2.5 years following approval of the first COVID-19 vaccine by the European Medicines Agency (EMA) in December 2020, resulting in an estimated 1.6 million lives saved during the period [3]. This achievement was possible thanks to a pre-existing landscape of vaccine R&D developers from the public and private sectors, emphasizing the need of continued investments in vaccine infrastructure to tackle current and future public health burdens. Here, we assessed the implementation and impact of the TRANSVAC projects and the lessons learned that are critical in the planning of future infrastructures.

## 2. Summary and Analysis of TRANSVAC Activities

The stated TRANSVAC mission is to “accelerate the pharmaceutical and clinical development of promising vaccine candidates by bridging the gap between academic research and clinical trials” [4]. This aim was primarily pursued through developing and offering scientific services and training to vaccinology researchers. The non-confidential results of the project were made publicly available through the EC’s Community Research and Development Information Service (CORDIS, https://cordis.europa.eu/, accessed on 20 October 2024).

### 2.1. Overview of TRANSVAC Services for Vaccine Candidates

Regardless of how well a consortium is organized and how strong its scientific capabilities are, a vaccine research infrastructure must build a portfolio of high-quality projects to succeed in improving public health. Since TRANSVAC’s mandate did not include developing its own vaccine candidates, it needed to raise awareness among vaccine developers in academia and small- and medium-sized enterprises (SMEs), encouraging them to submit applications to the consortium. This communication campaign promoted TRANSVAC as a provider of free, world-class vaccinology services.

**Figure 3:**
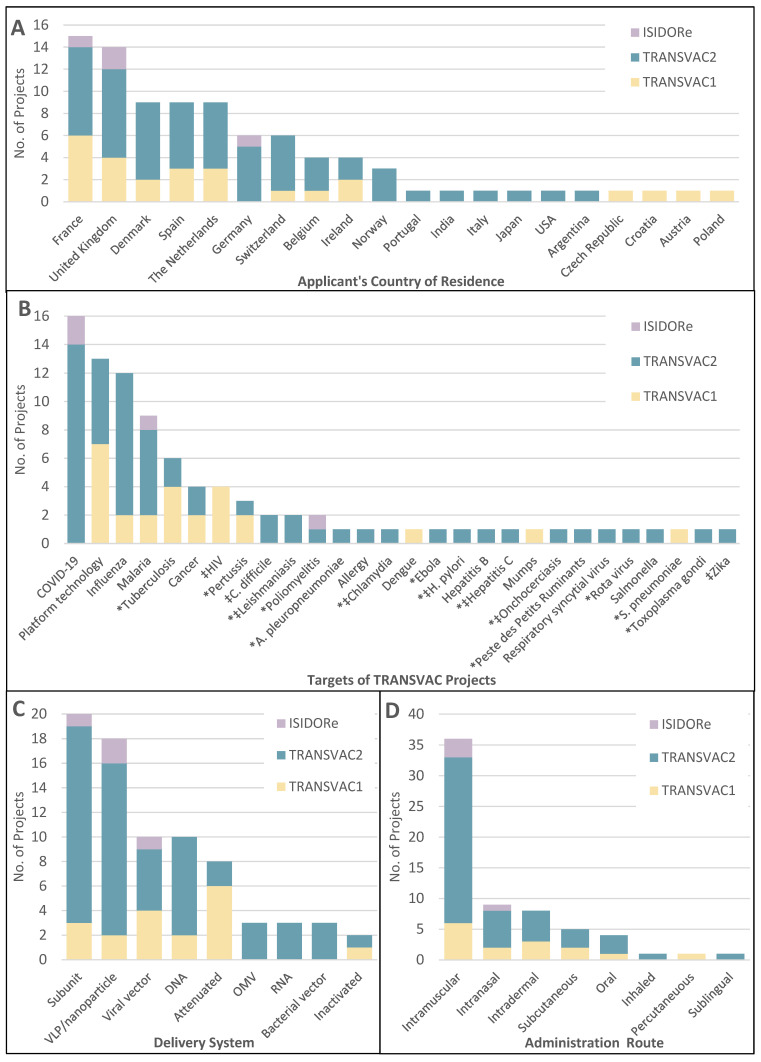
Distribution of TRANSVAC service projects. (**A**) Countries of residence of applicants awarded TRANSVAC projects. (**B**) Disease targets of TRANSVAC service projects. * Diseases not included in Vaccines Europe’s 2023 pipeline review [5]. ‡ Diseases with no approved vaccine currently available. (**C**) Delivery systems employed by TRANSVAC-supported vaccine candidates. Fifteen projects were omitted from the figure due to being too early stage (e.g., antigen identification), or platform-based, and three projects included multiple candidate delivery systems. (**D**) Intended administration route of TRANSVAC vaccine projects. Twenty-nine projects were not included as an administration route was not decided or unclear, and five projects involved hybrid protocols (e.g., prime-pull strategies) or compared multiple administration routes.

Over the years, TRANSVAC built a large network in the international vaccine community. A total of 161 applications for scientific services were received from developers based in 24 countries across Europe, Asia, North America, and South America. Eighty-nine of these applications were approved following the evaluation by panels of independent experts. The selected projects supported applicants from 20 different countries; 70% of approved projects came from EU member countries, and only 4 were from applicants outside the European continent (Figure 3A). The goals of these approved TRANSVAC projects were diverse, targeting 25 different human or animal pathogens plus cancer, allergies, and 13 ‘platform technology’ projects (Figure 3B). Activities in this last category did not focus on a particular pathogen but sought to generate novel vaccinology tools, primarily by developing and characterizing novel adjuvants and delivery systems.

Notably, 14 of the TRANSVAC pathogen targets were not addressed by any of the known vaccine candidates in the clinical development pipeline of Vaccines Europe members, based on a 2023 internal review of the portfolios of this group of 15 major European vaccine companies [5]. Several of these 14 targets were infectious diseases that have no currently approved vaccines (leishmaniasis, hepatitis C, *Helicobacter pylori*, onchocerciasis, and chlamydia). This highlights the importance of TRANSVAC in developing new or improved vaccines in areas that are neglected by pharmaceutical companies. The most common target of recent TRANSVAC projects was COVID-19, largely due to the provision of significant earmarked funding and dissemination support from the European Union for COVID-19-specific vaccine research and development projects. This support enabled TRANSVAC2 to tackle the pandemic crisis while still advancing projects focused on other public health needs. TRANSVAC2 supported a total of 18 COVID-19-focused projects, a remarkable number for a pathogen that only emerged in late 2019, midway through the project.

The delivery systems utilized in these projects were also diverse (Figure 3C). Half (38/77) of the classified vaccine candidates used antigen subunits, either unpackaged or within virus-like particle (VLP) or nanoparticle formats. Vector systems were used by 20% (16/77) of the candidates including viral vectors, bacterial vectors, and the relatively novel outer membrane vesicle (OMV) platform. A total of 17% (13/77) were either DNA- or RNA-based, with DNA more commonly used. For purposes of this classification, nucleotides packaged in nanoparticles were classified as DNA or RNA vaccines and not as VLP/nanoparticles. However, there has been increased interest in RNA-based vaccines since the success of the SARS-CoV-2 vaccines Spikevax and Cominarty, and providing more services in support of these vaccines should be a focus for future vaccine infrastructure.

The intended administration routes were also compared with TRANSVAC projects in which this information was known (Figure 3D). Most vaccines in use today are parenteral, injected either intramuscularly or subcutaneously. For reference, of the vaccines currently licensed by the U.S. FDA as of this writing, 92% are parenteral, with mucosal vaccines making up 8% [6]. However, mucosal vaccination is increasingly seen as an important method to increase the immune response at mucosal surfaces and better block the initial establishment of infections. While mucosal vaccines were also a minority of TRANSVAC projects, they were better represented at 23% (15/65) of candidates and include intranasal, oral, inhaled, and sublingual administration. This improved representation compared with established vaccines reflects a growing interest in mucosal vaccines among developers and TRANSVAC’s offerings of high-quality mucosal adjuvants and animal models to researchers.

#### 2.1.1. Therapeutic Vaccines

While most TRANSVAC-supported projects focused on prophylactic vaccines that provide protective immunity, the consortium also supported several therapeutic vaccine candidates. This class of vaccines is administered after infection or disease onset to stimulate the patient’s immune system to more effectively counter the threat. Therapeutic vaccines have the potential to address many of the chronic diseases that represent an increasing proportion of the public health burden [7]. Therapeutic vaccine projects in TRANSVAC targeted diseases such as hepatitis B, acquired immunodeficiency syndrome (AIDS), COVID-19, leishmaniasis, cancer, and the animal disease peste des petits ruminants (PPR), also known as ovine rinderpest.

#### 2.1.2. Interaction with the Vaccine Industry

**Figure 4:**
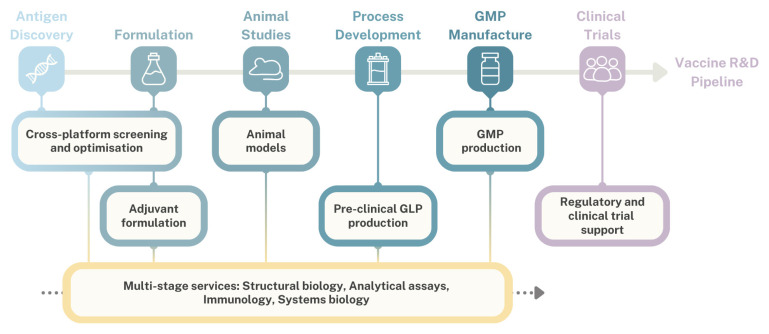
TRANSVAC scientific services provided along vaccine development pipeline.

While most applications for TRANSVAC scientific services (Figure 4) came from universities or public research institutions, approximately one quarter of the approved TRANSVAC projects went to private companies. Most of these private developers were classified as SMEs. These smaller biotechnology companies are critical players in the vaccine industry, and are often responsible for translating innovations and academic discoveries into public health solutions. However, they often have limited capacities and resources compared to larger multinational pharmaceutical companies and may lack sufficient preclinical capabilities needed to develop, optimize, and validate a vaccine candidate.

TRANSVAC was well-positioned to address these needs and sought to develop close interactions with the vaccine industry including industry representatives in advisory roles to ensure that the consortium met the user needs. Interest in TRANSVAC from industry grew over time, with private developers increasing from 11% of projects in TRANSVAC1 to 37% of projects approved in the second half of TRANSVAC2. This growth demonstrates an increased awareness of TRANSVAC and its growing reputation as a reliable partner for vaccine developers in the commercial biotech sector. This trend shows that, over time, a vaccine research infrastructure can become a trusted partner for SMEs, providing access to scientific facilities and expertise that are often unavailable internally in smaller firms.

#### 2.1.3. Developer Feedback

**Figure 5:**
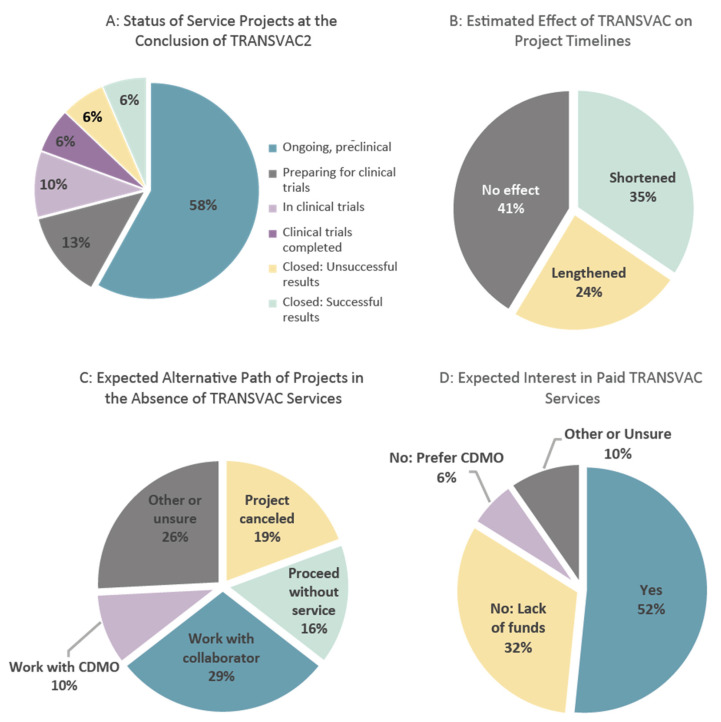
Results of the TRANSVAC scientific services user survey.

Each developer granted TRANSVAC support was asked to provide feedback on the provided services following the completion of their project. Additionally, at the conclusion of TRANSVAC2, a follow-up survey was conducted among all developers who were awarded services during the entirety of TRANSVAC1 and TRANSVAC2. Feedback was received from 31 respondents, providing insights into the impact of TRANSVAC services and the trajectory of projects from the vaccine developers’ perspective. Overall, the developers were pleased to have worked with TRANSVAC, rating the overall experience an average of 4.4 on a scale of 1 to 5 (where 1 was ‘poor’, and 5 was ‘excellent’). Of the 31 projects included, at the time of the survey, over half were continuing in pre-clinical studies (Figure 5A). Four had been terminated (half with successful results, the other half following unsuccessful findings), and another nine were either preparing for or had entered clinical trials. Developers who first applied for TRANSVAC services in 2018 or earlier were approximately one third of all respondents, but were responsible for six of the seven projects that had been terminated or completed clinical trials at the time of the survey. This suggests that at least 5 years are needed to assess the outcomes of most TRANSVAC projects.

While TRANSVAC aimed to accelerate the developmental process, respondents had mixed assessments of the effect of TRANSVAC on project timelines (Figure 5B). This metric, while subjective, reflects the potential for improving the service turnaround time in future TRANSVAC infrastructure. One strategy to improve this area would be a focus on streamlining the evaluation process and subsequent legal negotiations between TRANSVAC facilities and developers, as these processes often delayed the start of services. However, it will likely always be difficult for the academic partners to achieve turnaround times comparable to contract development and manufacturing organizations (CDMOs) as they are primarily organized as research organizations rather than pure service providers. Rather than quickness and consistency, the value of TRANSVAC services lies in their uniqueness, high-quality, and adaptability to specific needs. Indeed, based on the survey results, TRANSVAC services did not compete directly with CDMOs. When asked what developers would have done in the absence of TRANSVAC, only 10% would have contracted with a CDMO (Figure 5C). Similarly, most respondents expressed interest in paid TRANSVAC services if free services were unavailable, with only 6% of respondents preferring CDMOs over such a model (Figure 5D).

While developers were generally satisfied with the offered range of scientific services, one repeated request was for expanding the GLP/GMP production support. The need of the vaccine community for increased access to affordable manufacturing is a long-standing issue (Jungbluth et al., 2022) [2], but incorporating production into future vaccine infrastructures must be carried out carefully due to the high costs.

### 2.2. Development of TRANSVAC Services to Support Vaccine R&D

One of the principal advantages of combining multiple facilities under the umbrella of the TRANSVAC consortium was combining complementary services from across the TRANSVAC network into a single distributed infrastructure (Figure 4). For instance, an antigen might be generated and purified at a preclinical production facility, sent to adjuvant specialist partners for formulation, and then transferred to an animal facility for immunological studies. More details on the specific services offered under each of the categories in Figure 4 are available on the TRANSVAC website (https://www.transvac.org/vaccine-development-services, accessed on 15 October 2024). In total, fifteen TRANSVAC2 projects involved multiple service providers, and six projects received support from ≥3 TRANSVAC2 institutions, with smooth handovers from one TRANSVAC facility to the next as the projects progressed along the developmental pipeline.

As detailed below, the TRANSVAC scientific services were continually developed to ensure that the consortium’s offerings remained cutting-edge throughout the lifetime of the project. These efforts provided novel tools for the vaccinology community and improved coordination between TRANSVAC partners. Below, we highlight some of the key services and tools that were generated during the project and subsequently offered to the research community.

#### 2.2.1. Antigen Characterization and Selection

While most applicants approached TRANSVAC with selected antigen(s) and a lead vaccine candidate, some early-stage projects required assistance assessing and down-selecting potential target antigens. For the earliest projects, service began by comparing the expression of potential antigens in a variety of expression platforms including viral vectors, bacterial, yeast, plant, insect, and mammalian systems, available at TRANSVAC facilities in Frauenhofer IME, iBET, SSI, and BPRC. This was enabled by the creation of a central database of the available vectors, tools, and protocols to facilitate the testing of multiple expression systems for antigens across multiple institutions. This database and close collaboration between TRANSVAC members allowed the comparison of multiple candidate antigens and expression platforms with respect to protein yield and stability before moving to the small-scale production of pre-clinical grade material for the next steps in the development pipeline.

Following the initial production, numerous TRANSVAC services were offered to assist with the assessment of an antigen’s structural, biochemical, and immunological properties. Methods included analysis of the binding interactions of vaccine candidates with antibodies or ligands via cross-linking mass spectrometry (MS) or surface plasmon resonance (SPR) and the analysis of immune responses, both in vivo (transcriptomics, flow cytometry) and ex vivo (Luminex analysis of whole blood). Standardized operating procedures (SOPs) were made publicly available (https://cordis.europa.eu/project/id/730964/results, accessed on 15 October 2024). These new and optimized protocols were used in numerous TRANSVAC projects in support of antigen selection and evaluation. Such early-stage antigen selection and assessment capabilities position TRANSVAC, or a similar future research infrastructure, to not only support external projects, but also to potentially initiate novel vaccine candidates internally given sufficient financial resources.

#### 2.2.2. Adjuvants

Academic groups and private companies often face difficulties accessing safe, effective, and well-characterized adjuvants, as many adjuvants are either proprietary (and only available through complex licensing agreements) or lack sufficient validation. TRANSVAC was notably instrumental in contributing to the initiation in 2010 of the Vaccine Formulation Laboratory (VFL) at the University of Lausanne, Switzerland [8], which has now become the Vaccine Formulation Institute (VFI), an independent company of 30+ staff based in Geneva, Switzerland. VFI acts as a global center of expertise in adjuvants and formulation, facilitating the open-access of adjuvant technologies to the vaccine community, especially in low- and middle-income countries. Since 2010, VFI has been developing a portfolio of clinically relevant, open-access adjuvants based on liposomes or emulsions, such as Sepivac SWE™ (now commercialized by Seppic, France), SQ, LQ, and LMQ adjuvants, which have all been provided for formulation studies and immunogenicity testing as part of the TRANSVAC services.

In addition to these successes, TRANSVAC conducted other thorough, collaborative studies to address gaps in the adjuvants available to developers. TRANSVAC2 supported comprehensive studies of novel adjuvants including a detailed characterization of the CD8+ T-cell activating adjuvant CAF09b at the Statens Serum Institut (SSI) [9] and the mucosal/parenteral adjuvant cyclic di-adenosine monophosphate (CDA) at the Helmholtz-Zentrum Fur Infektionsforschung (HZI) [10,11]. Additionally, a novel Neisseria meningitidis expression system, developed for the production of an improved detoxified LPS adjuvant, was further optimized and characterized at Intravacc [12]. All of these efforts have improved the pool of available, validated adjuvants, and were among the most-requested services from external developers. The resulting adjuvants were utilized both in these services and in internal collaborative projects between TRANSVAC members including cross-species studies of vaccine adjuvants to assess the predictability of animal models [13,14,15].

#### 2.2.3. Immunization Routes

Alternate routes to intramuscular vaccination offer advantages including a reduction in vaccine hesitancy due to a fear of needles and potential for an improved mucosal response, leading to a better reduction in pathogen transmission [16]. TRANSVAC members contributed to advancing mucosal vaccine science on several fronts including the continued development, characterization, and offerings of the CAF09b and mucosal CDA adjuvants described above, and the generation and use of relevant animal models allowed for testing of the mucosal vaccine candidates and prime-boost immunization strategies. These collaborations resulted in numerous studies, both with external developers and between TRANSVAC partners; several of these mucosal projects resulted in publications [11,13,17,18,19], although the findings of most projects with external partners are still unpublished as research is still ongoing.

To review the future of alternative means of aerosol immunization, a 2-day workshop at the Stichting Wageningen Research Institution in the Netherlands was organized in 2023 by TRANSVAC and VetBioNet (https://vetbionet.eu/, accessed on 15 October 2024), another EU-supported research infrastructure that offers access to specialized animal models and high-containment facilities [20]. The workshop brought together various experts in the field to discuss the currently available different aerosol administrations in animal models. These represent enduring contributions to the field and demonstrate the potential of a vaccine infrastructure to develop novel therapeutics through the coordination of various institutions with complementary expertise and technologies.

#### 2.2.4. Vaccine Manufacturing Support

Optimizing and scaling the production, purification, and formulation of GLP/GMP-grade antigenic biomolecules or vectors, and sourcing large quantities of adjuvant materials are significant challenges that require specialized infrastructure and expertise. Improving the speed, capacity, and flexibility of European vaccine production has often been identified as a critical need in the field [2,21]. In addition to improving manufacturing access, there is also a need to invest in more flexible, decentralized manufacturing systems that can be rapidly scaled and adapted such as mRNA-based platforms.

TRANSVAC also provided support for a range of antigen expression platforms that included early screening/optimization, preclinical GLP, and GMP production. Specifically, TRANSVAC partners iBET and Genibet worked with vaccine developers to create and optimize processes for the transition and scale-up to GLP/GMP-compliant production, and VFI provided the GLP/GMP-compliant adjuvants. However, despite these activities, TRANSVAC’s direct involvement in vaccine manufacturing has been relatively limited due to the high cost of production services and support for GMP manufacturing, especially for vaccines without significant financial backing, which remains an unmet need of many developers.

### 2.3. Trainings in Vaccinology

TRANSVAC developed a specialized training program comprised of courses covering the entire process from vaccine R&D to licensure (Table 2), which was provided to the vaccinology community. The training was offered by TRANSVAC partner institutions across Europe free of charge to further strengthen the European and global vaccine landscape. This training filled a key gap, as many vaccine researchers and developers lack practical pathways to acquire specific skills that are not present within their institutions. Most TRANSVAC courses last 2 or 3 days, each providing in-depth hands-on training from experts on a specific vaccine-related topic without requiring an extended leave from the participants’ normal work duties, which makes them ideal for researchers needing to learn a particular skillset.

The foundation for the TRANSVAC training program was laid during TRANSVAC1 with the development of a 5-day course entitled ‘Practical approaches to vaccine development’ by the Vaccine Formulation Laboratory (VFL), now the Vaccine Formulation Institute (VFI). This first TRANSVAC course was held in 2012 and run again in 2013 with significant demand from the scientific community, with 62 applications received for 30 available spots. The courses were well-received, and following this success and the clear need for vaccine-related training, additional budget and resources were invested in TRANSVAC2 toward the development of a full vaccinology training program of 14 different courses covering the breadth of the vaccine development pipeline. While courses were held in-person, except for several that were moved online due to the COVID-19 pandemic restrictions, TRANSVAC2 member EATRIS subsequently developed their course ‘Regulatory aspects of vaccine development’ into a freely accessible eLearning module to provide the community with barrier-free access to the course content (https://eatris.eu/transmed-academy, accessed on 15 October 2024).

Some courses provided expertise in specific techniques while others discussed preclinical and clinical topics (Table 2). The latter category saw the most demand; the three most-requested courses were ‘Adjuvants and Vaccine Formulations’, ‘Regulatory Aspects of Vaccine Development’, and ‘Systems Biology of Vaccinology’, which averaged at least twenty-eight applications per call whereas more technical courses received relatively less demand. However, as the technical courses were also low capacity to ensure that all trainees received proper hands-on time with the techniques, even these courses received more applications than available spots, demonstrating a continued need for these training courses. Indeed, most courses were held twice over the course of TRANSVAC2, and all except two training sessions received increased demand the second time they were held, with the number of applications increasing by 69% on average in the second round.

#### 2.3.1. TRANSVAC Trainee Demographics

**Figure 6:**
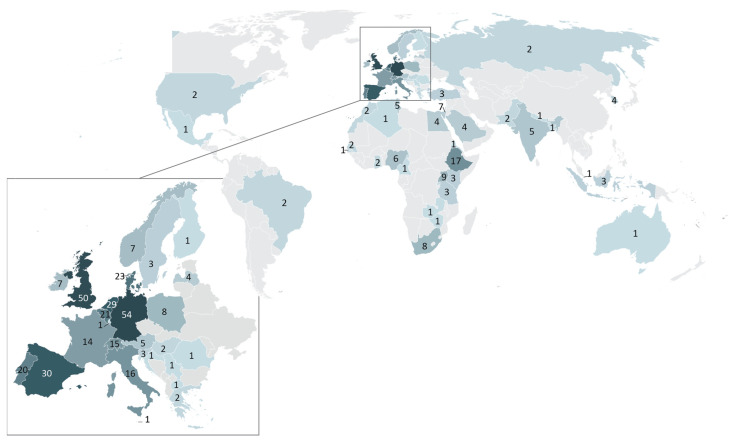
Distribution of applicants to TRANSVAC training events. Numbers correspond to the number of applications to TRANSVAC training events from applicants residing in each country.

A total of 427 unique applicants submitted a total of 718 applications to TRANSVAC2 courses, with many trainees applying to multiple courses. Despite the lack of travel funding available from TRANSVAC2, 40% of applicants resided outside the EU including 87 applicants (20% of total) from low- and middle-income countries (LMICs), as currently defined by the OECD. Seventy-seven percent of these LMIC applicants were based in Africa, with 87 training applicants from 17 African countries (Figure 6). This indicates a particular need for vaccine-specific training, especially across Africa.

Due to the limited capacity of training events, a standardized evaluation process was used to select applicants, with an overall acceptance rate of 59% (426 selected applications). Notably, LMIC applicants had an average acceptance rate of only 38%. This difference can be in part attributed to TRANSVAC2’s directive to give preference to applicants from EU member countries or associated countries, or who are involved in projects funded by the EU or the European and Developing Countries’ Clinical Trials Partnership (EDCTP). However, some of the acceptance rate gap is also likely to be due to the lower evaluations of the LMIC applications by the reviewers. This discrepancy should be considered by future programs to ensure that developers from regions with less opportunity to build experience and strong resumes can still access the necessary training.

The trainee backgrounds varied widely. Twenty percent of the applicants were PhD students, and approximately 50% had already earned their PhDs. The remaining 30% came from a wide variety of backgrounds including medical students, doctors, veterinarians, research technicians, and research assistants. While most applicants were from universities or public research organizations, approximately a quarter of applicants were employed by private institutions, and a small number worked within governmental agencies. The applicants also represented a wide range of the immunology community in terms of seniority, from master’s students and research technicians to senior scientists, group leaders, and department heads. From this analysis, there is evidently a broad demand for vaccinology training with respect to the trainees’ geography and background.

#### 2.3.2. Outlook for TRANSVAC Training Courses

Building on the success of the TRANSVAC2 training courses, the training providers reorganized themselves as the ‘TRANSVAC Academy’ in 2024. This independent training program utilizes a combination of support from donors, such as the Coalition for Epidemic Preparedness Innovations (CEPI), and registration fees to continue providing vaccinology courses (https://www.transvac.org/transvacacademy-training-courses, accessed on 15 October 2024). The program builds on the lessons learnt from TRANSVAC2 and includes new and modified courses with an additional focus on expanding access to participants from LMICs. This effort currently includes dedicated scholarships for LMIC trainees provided by CEPI and holding select courses outside the EU.

Additional vaccinology training courses and outreach developed and tailored toward including healthcare workers, regulators, teachers, and media members would help to fully address the public health needs. Such activities could address the broader issues of vaccine-related communication and vaccine hesitancy [22]. Confidence in vaccine safety and importance declined across Europe from 2020 to 2022, the period covered in the most recent EU Public Health report, reversing the previous gains and highlighting the need for improved public engagement [23]. Healthcare workers are particularly important in these efforts, and a significant percentage of nurses and physicians throughout Europe continue to report skepticism of the official vaccine recommendations [22]. Numerous groups involved in such social science topics and outreach could be included in such an effort including the VACCELERATE project [24], the Sonar-Global network (https://sonar-global.eu/, accessed on 15 October 2024), and the Vaccine Confidence Project (https://www.vaccineconfidence.org/, accessed on 15 October 2024).

## 3. Future Perspectives and Conclusions

Europe has a long tradition in vaccine research, and some of the world’s largest commercial vaccine manufacturers are based in Europe, with a substantial collective capacity and knowledge in vaccine discovery, development, and manufacturing. Smaller European biotechnology companies and academic institutions have also been driving forces for innovation in the field. Despite the significance and successes of the vaccine sector, continued investment and coordination are needed to build and maintain a healthy, competitive European vaccine R&D landscape that can address the public health needs.

In designing the future of Europe’s vaccine research infrastructure, other established research networks must be considered to ensure cooperative and complementary relationships rather than competition or redundancy. In practice, TRANSVAC often directly provided services to support other research consortia such as the EC-funded TBVAC2020 (Grant agreement ID 643381) and Prevent-nCoV (Grant agreement ID 101003608) projects, or contributed to collaborations that resulted in new projects such as Vax2Muc (Grant agreement ID 101080486, https://www.vax2muc.eu, accessed on 15 October 2024), SAPHIR (Grant agreement ID 633184), and the VACCELERATE project [24].

Established networks were also included as full RI partners; in TRANSVAC2, this was accomplished by incorporating Instruct-ERIC, a network providing access to advanced structural biology facilities to identify and characterize antigens. At the other end of the development pipeline, the European Infrastructure for Translational Medicine (EATRIS) and the European Clinical Research Infrastructure Network (ECRIN) were also members of TRANSVAC2 to provide support on regulatory issues and smooth the transition to clinical trials.

This concept was expanded in ISIDORe, forming a consortium of 17 RIs focused on pandemic preparedness and response. In addition to ensuring regular communications and positive working relationships between the networks, allowing for coordinated readiness planning and threat responses, ISIDORe also simplifies the European research infrastructure landscape with a single access point for applicants. In this way, ISIDORe plays a critical and necessary role in harmonizing various actors in the field.

Despite its successes, ISIDORe alone is likely to be insufficient as a long-term replacement for a dedicated vaccine research infrastructure. ISIDORe is primarily for the coordination of independent research infrastructures and is most effective when the member RIs are strong and able to take actions outside the purview of ISIDORe, according to the needs of their particular fields. In the case of TRANSVAC, the needs of the vaccinology sector extend beyond ISIDORe’s mandate for pandemic preparedness and response. These needs include improving the existing vaccine arsenal and addressing diseases that do not represent pandemic threats but are nevertheless acute public health concerns (e.g., tuberculosis, shigellosis, Lyme disease, veterinary infectious diseases, and non-communicable diseases such as cancer and neurodegenerative conditions. Additionally, in terms of budget, ISIDORe funding is 40% higher than that of TRANSVAC2, but spread among six times the number of partner institutions (Figure 1). At such levels, it will be difficult to maintain all of the partner organizations as active long-term participants in the existing RIs. Dedicated funding would produce a more effective RI, better equipped to improve its available services, meet the developers’ needs, contribute to the ISIDORe consortium, and mobilize for future threats.

A key area where such a dedicated vaccine infrastructure could improve on past achievements is better support for the critical but expensive transition from preclinical vaccine research to toxicology and early phase clinical trials. A commonly cited barrier in the process is access to GLP/GMP-level vaccine manufacturing, and implementing more manufacturing support could play an important role in future research infrastructure. However, production has unique considerations compared with the rest of the preclinical pipeline. It is difficult to evaluate these late-stage projects alongside early-stage proposals due to the production’s far greater costs and different risk/benefit profiles than activities at earlier stages in the pipeline, and it involves different domain expertise to properly evaluate. Additionally, granting significant public funds to late-stage vaccine candidates, typically owned by for-profit companies, will likely require a heightened level of oversight and interaction with the funding agencies. Therefore, manufacturing support would best be provided by either a dedicated infrastructure or by including an additional budget and a parallel evaluation track within a TRANSVAC-like infrastructure.

Another obstacle facing vaccine development is the current regulatory landscape for therapeutic vaccines. Despite several amendments to the relevant statutes [25], it remains a consensus that the current guidance is insufficient. As the 2023 Vaccines Europe Pipeline Review states, “There is no established regulatory and access environment pathway for therapeutic vaccines” [5]. This need for clarity was also emphasized by interviewed developers in a gaps-and-needs analysis of the European vaccine landscape including suggestions to classify therapeutic vaccines as an immunotherapy and harmonizing regulatory guidance between the EMA and national frameworks while acknowledging the difficulties stemming from the different forms and uses of therapeutic vaccines [2]. This need for coordination, both within the therapeutic vaccine developer community and between developers, funders, and regulators, requires a long-term effort and could be facilitated by a vaccine research infrastructure.

This cooperation could improve the regulatory environment in several ways. First, instituting specific EMA guidelines for regulatory approval requirements for immunotherapies would assist in harmonizing the process at the European level. Vaccine developers could provide input to such guidelines as the output of workshop discussions. Additionally, regulatory bodies often lack specific expertise in therapeutic vaccines as the field is relatively new. Further dialogue with regulators, which could include workshops and increased participation in EMA meetings, could increase the regulators’ familiarity with this emerging field.

Alternative financial mechanisms have been proposed and explored in detail to produce a sustainable entity to carry on TRANSVAC’s mission and address these needs. A self-sustaining entity would need to provide vaccine developers access to scientific services without relying on public funding. Recommended business models for such a vaccine research infrastructure were recently published following an EC-supported design study by 25 European organizations involved in vaccine research and development including many TRANSVAC2 partners (TRANSVAC-DS, Grant agreement ID 951668) (Jungbluth et al., 2023) [26]. The proposed model would rely upon a combination of fee-for-service and equity stakes in supported vaccine candidates. Such a self-sustaining model could be a powerful solution but requires significant start-up capital and risk, and would necessitate additional financial assessments and arrangements when selecting projects to ensure long-term solvency. However, given the lack of public funding, such an infrastructure would have relatively limited ability to provide scientific services to developers with constrained budgets or with projects of significant potential health benefit but minimal profit expectations.

Without public funding, there would also be less resources for continued investment in the vaccinology offerings of an infrastructure. During TRANSVAC, internal research activities ensured that the consortium continually developed new, cutting-edge services that were offered to external developers. Further work is needed to continue addressing critical gaps and ensure that academics and SMEs can access the latest vaccine platforms and technologies such as formulation and manufacturing support for mRNA-based vaccines. As described above, during TRANSVAC, the research activities not only improved the infrastructure’s capabilities but generated and validated novel tools such as adjuvants, animal models, and assay protocols that are now available to the vaccine community beyond the horizons of TRANSVAC. Continuing to provide these resources as well as facilitating access to the needed scientific services and contributing to European pandemic preparedness and response efforts would be best enabled by a long-term commitment by European member states to support a sustainable and dedicated vaccine R&D infrastructure that meets the needs described here.

Such an infrastructure would be a pivotal step toward enhancing pandemic preparedness, enabling coordinated and continuous research efforts, thus ensuring readiness to address both pandemic threats and neglected diseases. If establishing a permanent infrastructure is considered not feasible, providing long-term and flexible financing to such initiatives might be an alternative. This would ensure their capacity to operate effectively, free from the constraints of short project cycles or the disruptions of shifting political priorities. On a global level, there has been a move toward more localized vaccine development and production capacity since the pandemic. Notably, the African Union aims to increase Africa’s vaccine production dramatically, from under 1% of the continent’s needs in 2022 to 60% by 2040, and similar efforts to improve vaccine security and self-sufficiency are underway across the world [27]. A vaccine infrastructure could assist by providing the new hubs with access to expertise, technology transfer, and training in vaccine R&D and production. If successful, this shift should ensure a more distributed and robust system for responding to pandemic threats.

The TRANSVAC projects successfully integrated participating institutions into a productive, goal-oriented, and successful research and development infrastructure. The projects demonstrate a viable path to combine leading European institutions into a vaccine infrastructure and provide developers with access to scientific services, technologies, and training. Despite the success and popularity of the effort, it is important to not only preserve the accomplishments, but to continue assessing how future infrastructure can better meet the needs of the vaccine field and the broader public.

## Figures and Tables

**Table 1:** Institutes involved in the TRANSVAC consortia *.

Institution	Country	TV1	TV2	ISIDORe	Primary Roles
Biomedical Primate Research Center (BPRC)	The Netherlands	X	X	X	Immunogenicity studies in NHPs
Department of Health (DH)/Medicines and Healthcare Products Regulatory Agency (MHRA)	United Kingdom	X	X	X	Optimization of immunoassays incl. Luminex and ELISpot
European Vaccine Initiative (EVI)	Germany	X	X	X	Coordination, regulatory advice
Helmholtz-Zentrum für Infektionsforschung (HZI)	Germany	X	X	X	Murine immunogenicity studies; development and formulation of mucosal adjuvants
Stichting Wageningen Research (SWR)	The Netherlands	X	X	X	Immunogenicity studies in ferrets and pigs
University of Oxford	United Kingdom	X	X	X	Antigen expression and production in viral vaccine vectors
London School of Hygiene and Tropical Medicine (LSHTM)	United Kingdom	X	X		Immunoassays incl. Luminex, ELISpot
Public Health England (PHE)/UK Health Security Agency	United Kingdom	X	X		Immunogenicity studies in ferrets
Vaccine Formulation Laboratory (VFL), University of Lausanne (UNIL)	Switzerland	X	X		Adjuvant formulation and characterization, training
Commissariat à l’énergie atomique (CEA)	France		X	X	NHP studies, immune analysis via mass and flow cytometry
European Advanced Translational Research Infrastructure in Medicine (EATRIS)	The Netherlands		X	X	Regulatory advice and resources
European Clinical Research Infrastructure Network (ECRIN)	France		X	X	Clinical trial support
Fraunhofer-Gesellschaft (Fraunhofer)	Germany		X	X	Antigen expression and characterization
GenIbet Biopharmaceuticals	Portugal		X	X	GMP production
Institut de Recerca i Tecnologia Agroalimentaries (IRTA)	Spain		X	X	Immunogenicity studies (hamsters, mice, ferrets, small ruminants, and swine)
Institut National de Recherche pour l’Agriculture, l’Alimentation et l’Environnement (INRAE)	France		X	X	Immunogenicity studies in rabbits and pigs
Instituto de Biologia Experimental e Tecnológica (iBET)	Portugal		X	X	Development and production of GLP material
Instruct-ERIC	United Kingdom		X	X	Structural biology incl. cryo-EM
Leiden University	The Netherlands		X	X	Metabolomics
Leiden University Medical Center (LUMC)	The Netherlands		X	X	Multiplex transcriptome profiling
Statens Serum Institut (SSI)	Denmark		X	X	Development and formulation of liposomal adjuvants; murine immunogenicity studies; protein expression/ GLP production
Universita degli Studi di Siena (UNISI)	Italy		X	X	Flow cytometry and sequencing assays and analysis
Vaccine Formulation Institute (VFI) (formerly VFL)	Switzerland		X	X	Adjuvant formulation and characterization, training
LIONEX Diagnostics and Therapeutics	Germany	X			Antigen expression and purification
Max Planck Institute for Infection Biology (MPIIB)	Germany	X			Functional genomics
Serum Life Science Europe (SLS Europe, formerly Vakzine Projekt Management)	Germany	X			Clinical trial support and project evaluation
Tuberculosis Vaccine Initiative (TBVI)	The Netherlands	X			Coordination
University of Regensburg	Germany	X			Genomic analysis
BIOASTER	France		X		-omics (metabolomics, proteomics, RNA-Seq)
Eidgenoessische Technische Hochschule Zuerich (ETHZ)	Switzerland		X		Systems biology modeling
Intravacc	The Netherlands		X		Proteomics; development and formulation of LPS adjuvants
Sclavo Vaccines Association (SVA)	Italy		X		Impact analysis

* Note: ISIDORe members who did not participate in previous TRANSVAC projects were excluded from the table. In the case of name changes, the most recent institute name was used and may differ from previous TRANSVAC documents.

**Table 2:** Summary of the TRANSVAC2 training courses.

Course	Organizer	1st Round	2nd Round	3rd Round	Total Applications	Selected Trainees
Clinical Vaccine Development and Biomanufacturing	University of Oxford	October 2018	September 2021	October 2022	58	33
Human and Veterinary Vaccinology	University of Oxford	November 2018	October 2021		39	29
Adjuvants and Vaccine Formulations	VFI	March 2018	March 2022	April 2023	155	40
Validity and Translational Aspects of Animal Models in Vaccine Research	SWR	March 2022	October 2022		50	47
Statistics for Vaccine Evaluation Program	BPRC	June 2019	March 2022	March 2023	40	20
Mass Cytometry	CEA	September 2019	April 2023		29	24
Flow Cytometry	CEA	September 2019	Cancelled		17	12
In Vivo Imaging	CEA	March 2020	April 2023		14	13
Process Development of Cell Culture Viral Vaccines	VFI/ Merck	September 2019	September 2021		41	23
Application of SPR Technologies in Vaccine Development and Manufacturing	Fraunhofer	October 2019	November 2021		27	18
Process Development and Scale-Up	Fraunhofer	October 2019	October 2021		49	27
Requirements for GMP Production	Fraunhofer	October 2019	November 2021		33	24
Systems Biology of Vaccinology	UNISI	June 2020	February 2023		56	36
Regulatory Aspects of Vaccine Development	EATRIS	November 2020	March 2022	March 2023	110	80
Total					718	426
